# Supplementary material for: Using Machine Learning to Develop a Fully Automated Soybean Nodule Acquisition Pipeline (SNAP)
Source: Plant Phenomics. 2021 Jul 28;2021:9834746. doi: 10.34133/2021/9834746 (PMC8343430; doi:10.34133/2021/9834746)
Supplement: Supplementary Materials — Figure S1: width and height of the annotated nodules. Figure S2: width distribution of the annotated bounding boxes for nodules from 30% of the dataset. Figure S3: aspect ratio distribution of the annotated bounding boxes for the nodules from 30% of the dataset. Figure S4: informative sample selection workflow. Figure S5: focal (classification) and regression (bounding box detection) losses during training of nodule detection network, RetinaNet, using input image scale 512, anchor scales 0.48, 0.67, and 0.86, and aspect ratios 0.85, 0.99, and 1.13. Figure S6: the UNet architecture used to develop the tap root detection model. Figure S7: training and validation losses (Jaccard loss) during the training of UNet for tap root detection. Figure S8: effect of input image scale on nodule detection in the test data. Figure S9: representative example of good nodule detection on a V5 growth stage soybean root. Figure S10: a rare example of high misclassification of image debris as nodules on a V5 growth stage soybean root. Figure S11: (A) input image, (B) SNAP-detected nodules, (C) segmented image with difficult-to-detect clusters of nodules, (D) 50 strongest SURF points on original grayscale image, (E) 50 strongest SURF points on masked grayscale image, and (F) samples of training image patches used for bag-of-features codebook generation. Table S1: sizes, aspect ratios, and range of anchor configuration. Table S2: comparison of average times taken to extract, wash, and image roots in this study with average times required to hand quantify nodules compared to SNAP Quantify. [file 9834746.f1.docx]

**Supplementary Materials**


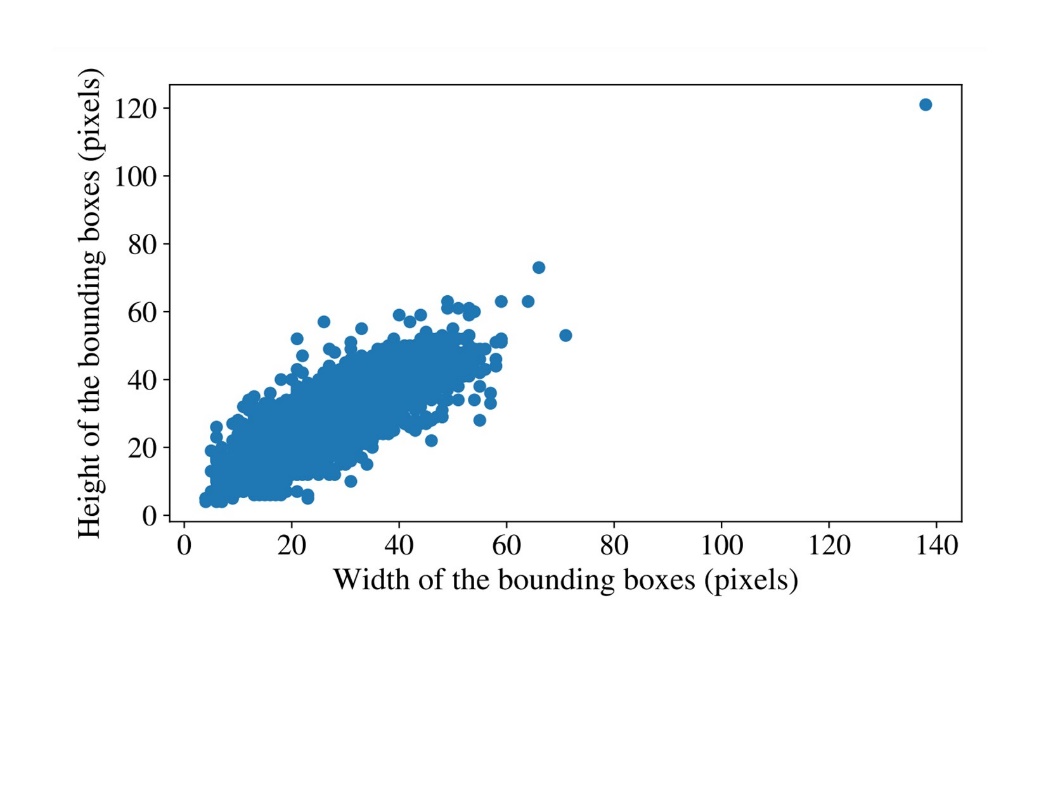


**Figure S1:** Width and height of the annotated nodules.

0

20

40

60

80

100

120

140

Width (pixels)

0.00

0.01

0.02

0.03

0.04

0.05

Density (Fraction)

**Figure S2:** Width distribution of the annotated bounding boxes for nodules from 30% of the dataset.

0

1

2

3

4

5

Aspect ratio

0.0

0.5

1.0

1.5

2.0

Density (Fraction)

**Figure S3:** Aspect ratio distribution of the annotated bounding boxes for the nodules from 30% of the dataset.

**512 x 512**

**512 x 512**

**512 x 512**

**512 x 512**

Facility Location


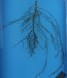

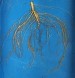

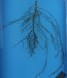

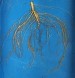

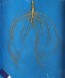

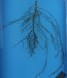

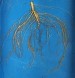

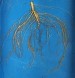

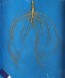

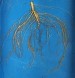


Flatten

W

R

**512 x 512**

**3**

**16**

**16**

**256 x 256**

**16**

**3**


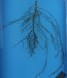

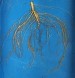

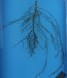

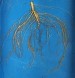

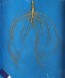

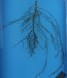

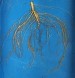

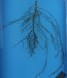

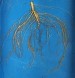

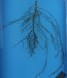

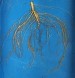

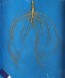

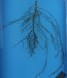

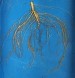


PCA


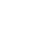

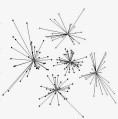


**16**

Bilinear interpolation

Conv 3x3, ReLU

Maxpool 2x2

Up sampling2x2

Conv1x1, sigmoid

**Figure S4:** Informative sample selection workflow. Latent features of the images (W) were obtained via dimensionality reduction using convolution autoencoder and principal component analysis (PCA) as shown in the box. A set of information samples (R) (with predefined size) were selected using a facility location based submodular function optimization.


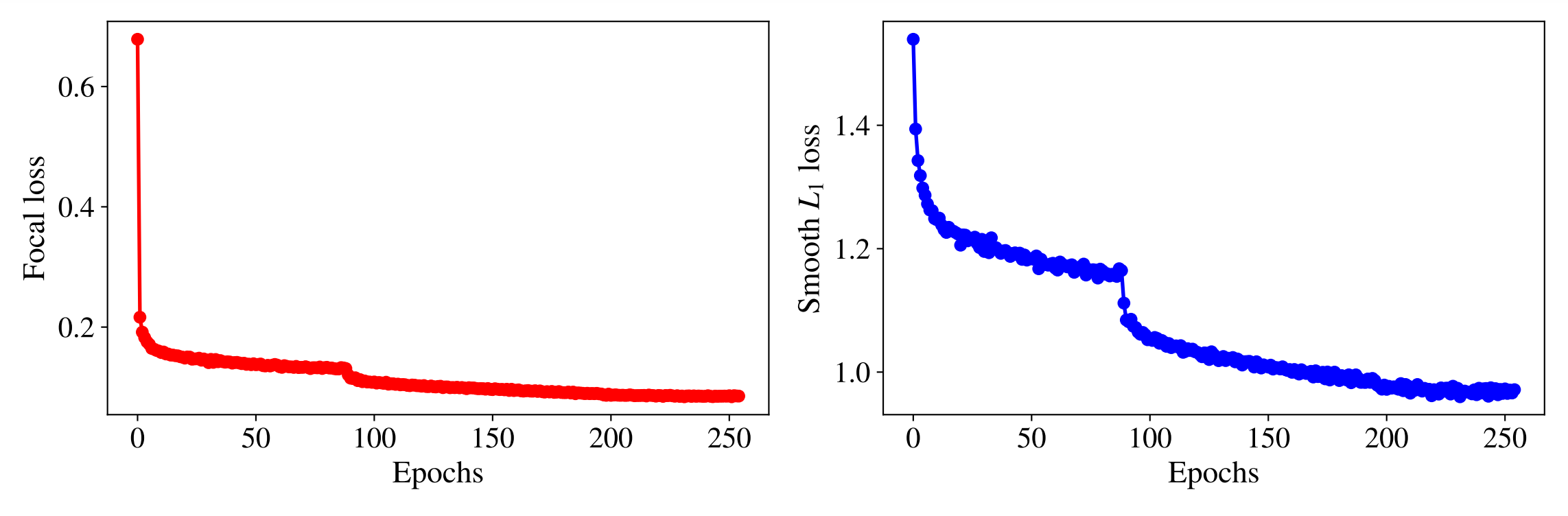


**Figure S5:** Focal (classification) and regression (bounding box detection) losses during training of nodule detection network, RetinaNet, using input image scale 512, anchor scales 0.48, 0.67, 0.86, aspect ratios 0.85, 0.99, 1.13.


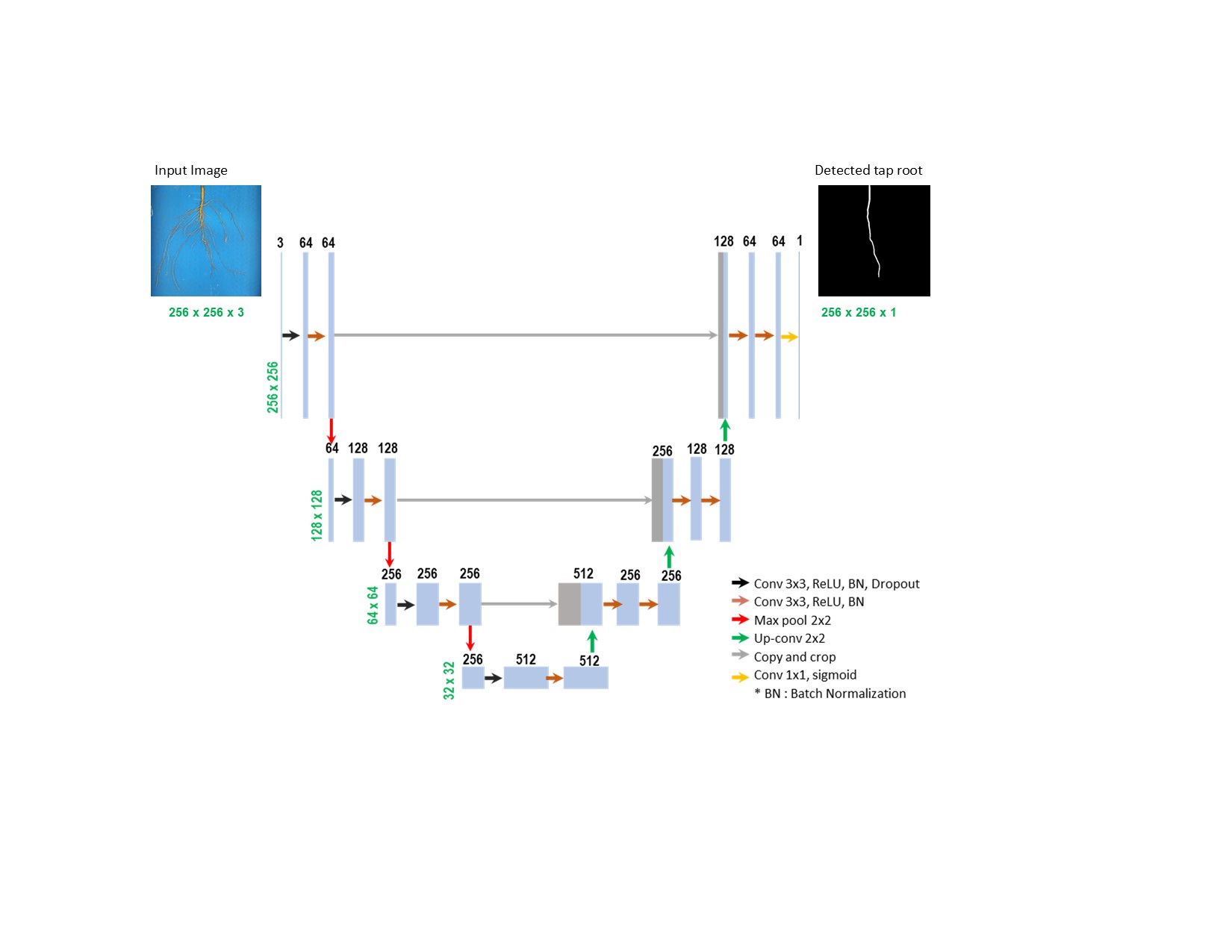


**Figure S6:** The U-Net architecture used to develop the tap root detection model. Each blue bar represents a multi-channel feature map. The number of channels is denoted on top of the bar, and width x height is provided on the left of the box. Gray bars are copied feature maps. Colored arrows denote the different operations as indicated in the lower right arrow legend.

0

25

50

75

100

125

150

175

epoch

0.4

0.6

0.8

1.0

1.2

1.4

Jaccard Index

training

validation

**Figure S7:** Training and validation losses (Jaccard loss) during the training of U-Net for tap root detection.


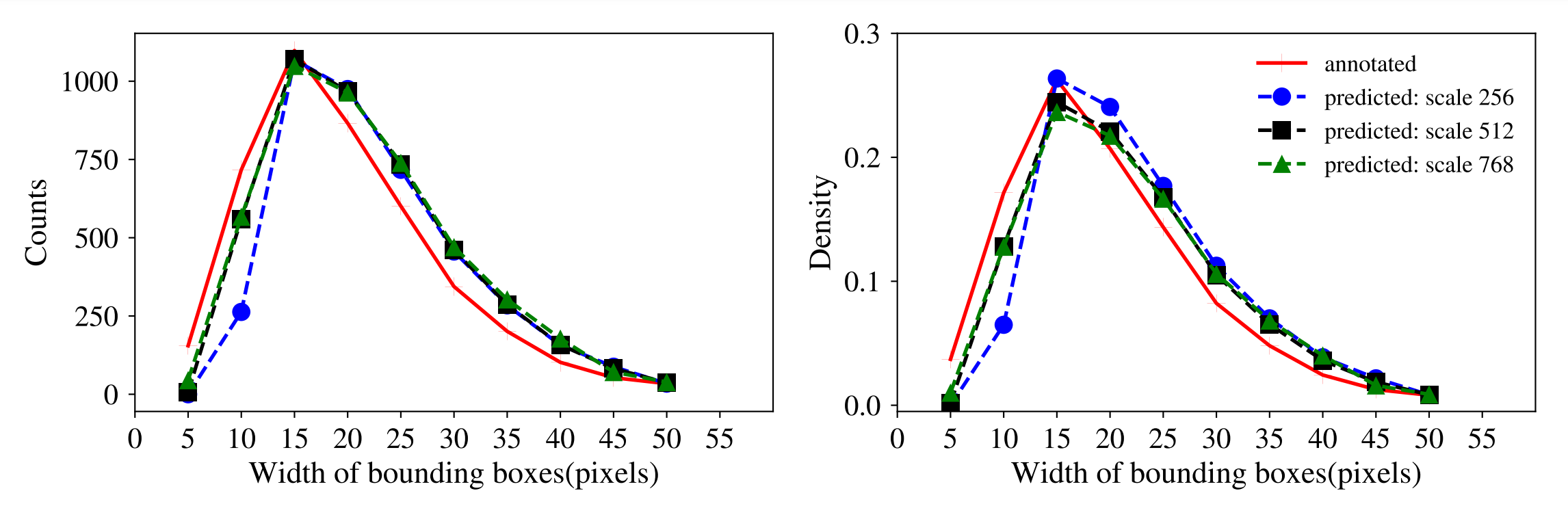


**Figure S8:** Effect of input image scale on nodule detection in the test data. The red solid line indicates the distribution of the annotated bounding boxes in the test data and the dotted lines are the distributions of the predicted bounding boxes at different input image scales.


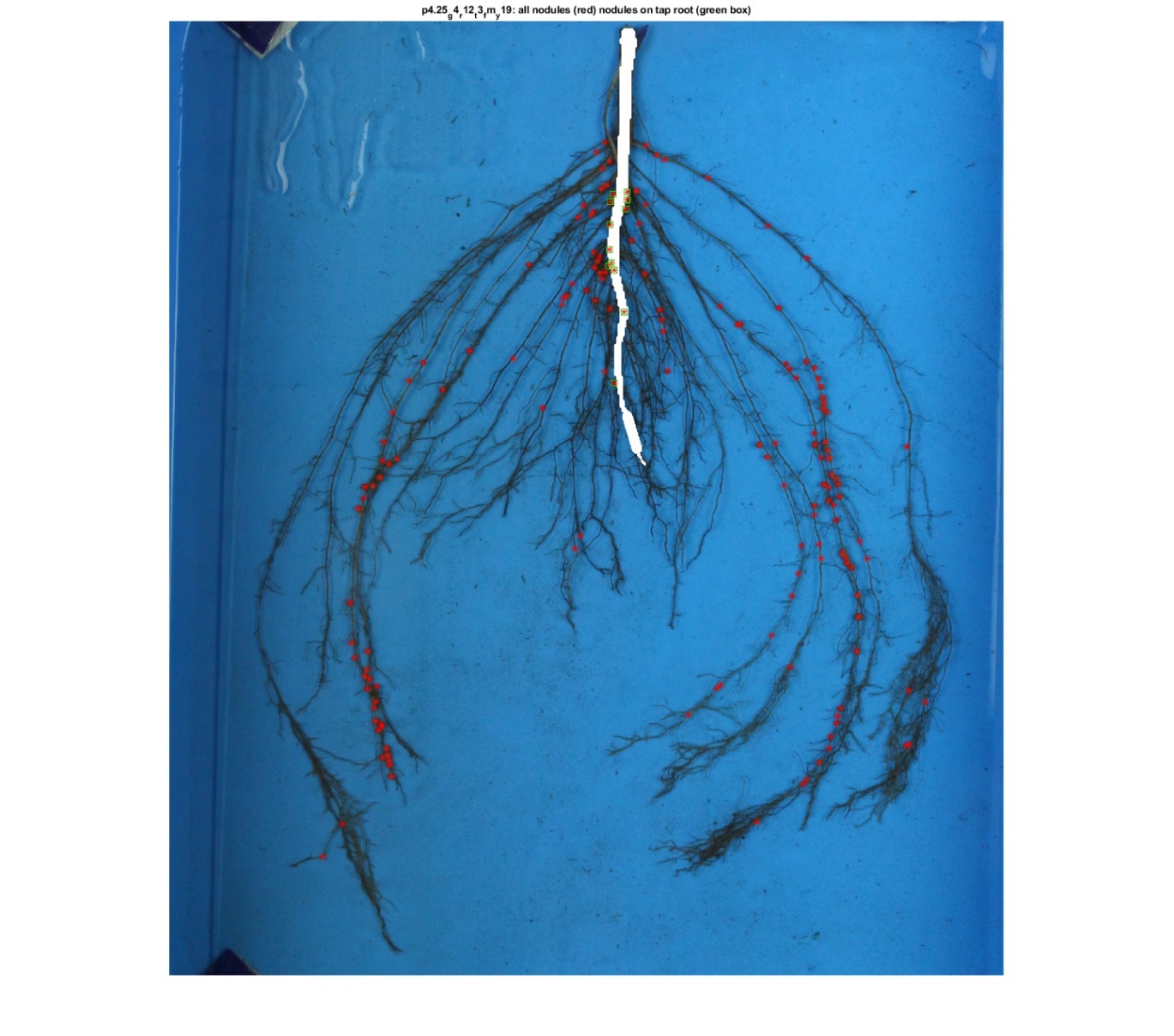


**Figure S9:** Representative example of good nodule detection on a V5 growth stage soybean root.


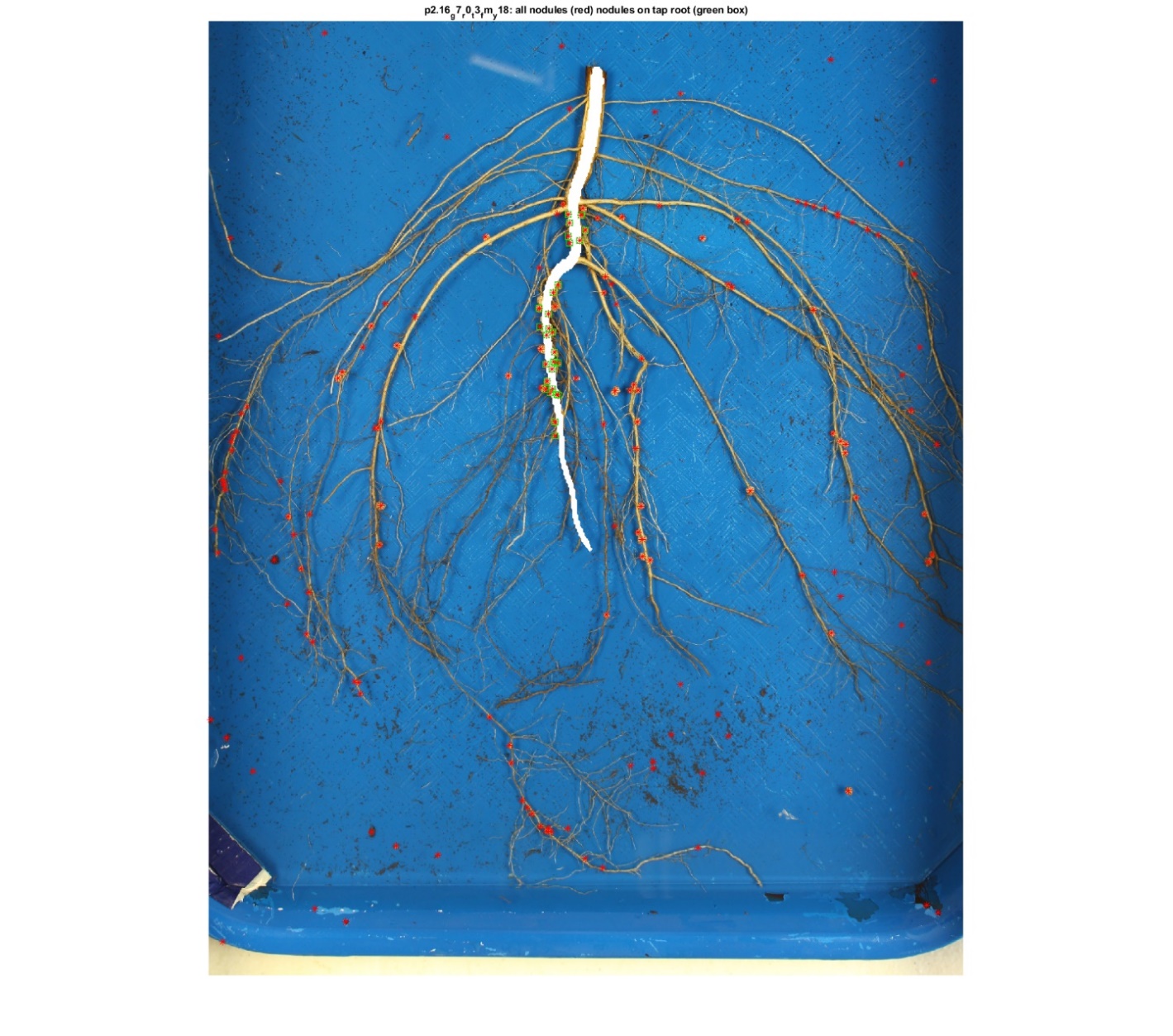


**Figure S10:** A rare example of high misclassification of image debris as nodules on a V5 growth stage soybean root. While most of the actual nodules were accurately identified, additional materials in the image were misclassified as nodules.

1.
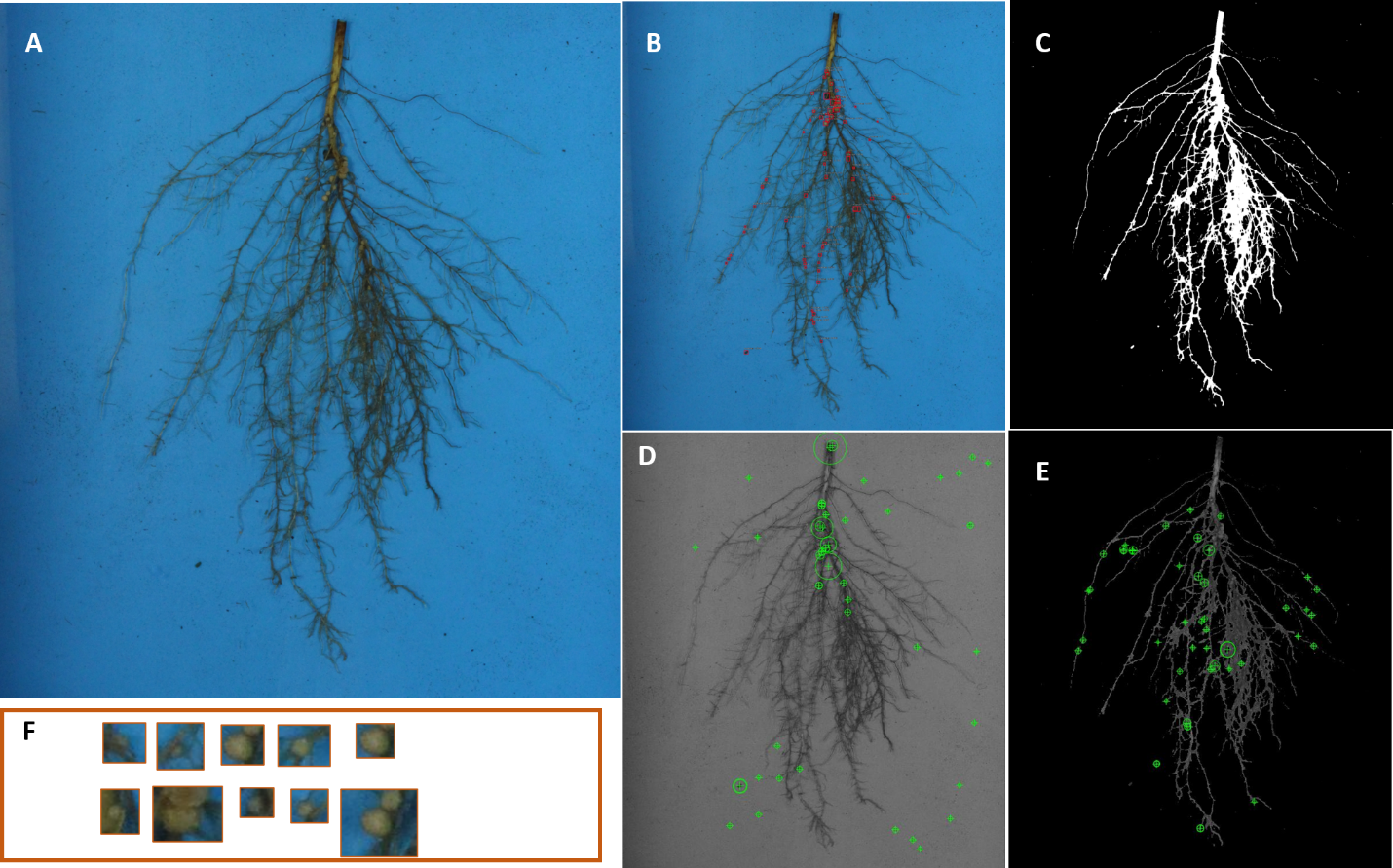


**Figure S11:** A) input image; B) SNAP-detected nodules C) segmented image with difficult-to-detect clusters of nodules D) 50 strongest SURF points on original grayscale image E) 50 strongest SURF points on masked grayscale image F) samples of training image patches used for bag-of-features codebook generation.

**Challenges with the traditional machine vision methods:**

We used several traditional machine vision methods to detect nodules on the root images. Those methods did not work for us. Here are a few examples based on a sample root image (shown in Figure S11) (A). Figure S11(B) depicts SNAP-detected nodules.

1. We attempted to use the algorithm described in [1], which includes segmenting the root from the background, skeletonizing the root, and detecting nodules based on specific morphometric and color features. For our dataset, heuristic color-based segmentation could delineate 90-95 percent of the root part of an image from the background, but the remaining part is extremely difficult to delineate with the heuristic method (Fig. S11 (C)). It was also challenging to obtain a faithful skeletonization of the overlapping and complex root (Fig. S11(C)).

1. We attempted to consider nodules as distinct from the rest of the root. We were hoping that it has distinct characteristics that will be picked up by the feature extraction method, faster and more robust features (SURF). Figure S11(D-E) depicts the top 50 strong SURF points (green points) on the original (gray scale) and masked gray scale images. Both distributions show that not all SURF points are nodules, and not all nodules are detected as SURF points.

1. We tried a bag of features strategy [2], which entails creating a feature codebook and then using sliding-window on the entire image to identify patches of the image where the features in the codebook match. However, we were unable to generate a good codebook of nodule features. For example, Fig. S11(F) depicts ten patches of images from our training data that were used to generate the codebook. SIFT descriptors were provided by only three patches.

**Table S.1:** Sizes, aspect ratios, and range of anchor configuration.

| **Method** | **Number of scales, number of aspect ratios** | **Anchor scales** | **Anchor aspect ratios** | **Coverage range** |
| --- | --- | --- | --- | --- |
| Default | 3, 3 | 1, 1.26, 1.59 | 0.5, 1, 2 | 32-813 |
| Optimized | 3, 3 | 0.4, 0.5, 0.625 | 0.69, 1.0, 1.43 | 13-508 |
| Normal Percentile | 3, 3 | 0.48, 0.67, 0.86 | 0.85, 0.99, 1.13 | 15-28(P2) … 245-440(P5) |
| Normal Percentile | 5, 5 | 0.39, 0.54, 0.67, 0.79, 0.95 | 0.79, 0.9, 0.99, 1.08, 1.2 | 12-485 |
| Normal Percentile | 7, 7 | 0.32, 0.47, 0.57, 0.67, 0.76, 0.87, 1.01 | 0.74, 0.85, 0.92, 0.99, 1.06, 1.14, 1.25 | 10-512 |

**Table S.2:** Comparison of average times taken to extract, wash and image roots in this study with average times required to hand quantify nodules compared to SNAP Quantify.

| **Growth Stage** | **Average time required in seconds for tasks:** | | | | **Factors of SNAP Improvement Over Hand Quantification** |
| --- | --- | --- | --- | --- | --- |
|  | **Extraction** | **Washing & Image** | **Hand Quantify** | **SNAP Quantify** |  |
| **V1** | 240 | 100 | 1500 | 90 | 16.6 x |
| **V3** | 360 | 128 | 2100 | 120 | 17.5 x |
| **V5** | 420 | 150 | 3000 | 150 | 20.0 x |

**Description of the libraries and frameworks in the development of SNAP**

The libraries and frameworks used in the development of SNAP are described in GitHub repository https://github.com/SoylabSingh/SNAP. However, for the sake of completeness, we have included an overview of the repository.

The SNAP software or repository is organized in three sections as presented in the manuscript.

Nodule detection: This section takes a root image as the input and draws bounding boxes around the detected nodules. The model was based on a RetinaNet on a ResNet50-FPN backbone. The model was trained using ~140 diverse root samples. The AP of the model on ~30 test samples was 0.62. The base code of this model was borrowed from <https://github.com/fizyr/keras-retinanet> (version 0.5.1, commit id 01737e6523c09df922d36edac332a68bcda4af90). We introduced anchor selection based on the natural size distribution of nodules.

Tap root detection: This section takes a root image as the input and provides a binary image of the tap root. The model was based on UNet. The model was trained on using ~40 diverse root samples. The Jaccard loss (index) on ~10 test samples was 0.5. This was based on [https://github.com/karolzak/keras-unet](https://github.com/karolzak/keras-unet.) (version 0.0.7, commit id 531f49fa8ad85ffbe1042ffa72f8c010ce329236)

Post processing: This section takes the outputs from the nodule detection and Tap root detection models as inputs and outputs the total number of nodules per image and number of nodules close to the tap root. The script is written in MATALB and it performs classical image processing to generate the outputs.

To install SNAP, please follow the instruction below:

1. Download the repo to local machine.
2. Download and install the latest version of anaconda. (<https://www.anaconda.com/products/individual>)
3. Create a new environment named "snap" using "snap.yml" (located in the downloaded repository) as:

$ *conda env create -f snap.yml*

Once the environment has been built, use the following to activate the environment.

$ *conda activate snap*

1. Install MATLAB 2018a (or newer) with image processing toolbox.

To use SNAP, please follow the instruction below:

To use the first two sections, open a jupyter notebook inside the snap environment and navigate to the desired section to open and execute the .ipynb

- To detect the nodules on the images in the sample_images folder run *nodule_dector_example.ipynb* notebook in the nodule_detection folder. The detected nodules on the images and corresponding locations can be found in sample_outputs/nodules.
- To detect the tap root on the images in the sample_images folder run *tap_root_detector_example.ipynb* notebook in the tap_root_detection folder. The detected tap root on the images can be found in sample_outputs/tap_roots.
- To identify the number of nodules on or near the tap root run *post_processing.m* using MATLAB in the post_processing folder. The detected nodules on the tap root can be seen in nodules_on_tap_root. The total number of nodules per image and number of nodules on the tap root can be found in *nodules_distribution.csv* in the sample_outputs/counts folder.
- To create a csv containing all of the unique nodules of a dataset across all of the roots, run *merge_csvs_nodule_dectection.ipynb* in the post_processing folder.

Supplemental References:

1. Lauren Remmler et al. “Standardized mapping of nodulation patterns in legume roots”. In: *New Phytologist* 202.3 (2014), pp. 1083–1094.
2. Nowak, Eric, Frédéric Jurie, and Bill Triggs. "Sampling strategies for bag-of-features image classification." *European conference on computer vision*. Springer, Berlin, Heidelberg, 2006.
